# Chapped Hands

**Published:** 1872-01

**Authors:** 


### CHAPPED HANDS

Are caused by having the hands a good deal in water, and going to the fire immediately after, as in washing dishes, cooking, etc., in very cold weather; the skin being softened by the water, and dried too rapidly by the fire, contracts and cracks; sometimes the fingers become so sore that sewing is impossible. The best plan of treatment is to wash the hands in the morning only, not to allow them to touch water the whole day after; then at bedtime put half a teaspoonful of sweet oil in the palm, and work it into the skin of the hands and fingers most thoroughly; put on a pair of thread or old kid gloves at bedtime, and wear them all night; there will be a wonderful relief of the soreness by the next morning, and a perfect cure in two or three days. If the hands must be in water more or less every day, then try the oil and gloves at night, using the same gloves without washing, all winter, if necessary, for they become saturated with the oil, and thus act more efficiently by keeping the skin soft all the time. A great many complicated mixtures are advised, but there is nothing equal to sweet oil or glycerine, used as above. The glycerine answers a good purpose, if worked into the hands every morning, just after they are washed for the day. Honey is said to be very good in this connection, but a tidy person recoils at the idea of having honey, or anything else sticking to the hands and fingers all day. Some persons advise, " Take equal parts of unsalted, fresh butter, mutton tallow, beeswax, and stoned raisins. Simmer until the raisins are done to a crisp. Strain, pour into a cup and let it cool, and rub it into the hands and lips before going to bed or going out into the wind." The only really beneficial ingredient in the above is the unsalted butter; and oil or glycerine are much better.

